# Human-anchored longitudinal comparison of generative AI with a bias-calibrated LLM-as-judge

**DOI:** 10.1371/journal.pone.0339920

**Published:** 2026-02-02

**Authors:** Thomas Wiese

**Affiliations:** SUNY Empire State University, New York, United States of America; Woldia University, ETHIOPIA

## Abstract

Service LLMs evolve without public changelogs, complicating reproducible evaluation. We present a preregistered human-anchored longitudinal study that tracks three major model families over ten weekly waves using a fixed prompt bank (N = 240) across six domains. Blinded human raters provided correctness judgments, and a bias-calibrated LLM-as-judge produced secondary pairwise preferences corrected weekly via a Bradley–Terry model. Mixed-effects modeling and change-point detection (PELT with MBIC penalty) identified significant service drift patterns. Results show divergent stability trajectories among models: one stable, one improving, and one degrading mid-study. Judge calibration increased agreement with humans (τ = 0.59–0.68) while reducing volatility. Safety metrics co-varied with drift events, suggesting behavioral shifts rather than confirmed causal changes. All data, prompts, rubrics, and parameter configurations are provided in supporting files S1–S6.

## 1. Introduction

Large language models (LLMs) offered as services are increasingly embedded in scientific, educational, and commercial applications [1]. A critical problem facing users is the opaque nature of their development cycles, which obscures changes to model parameters, training data, and alignment strategies. This opacity leads to service drift, the phenomenon where a model’s behavior and performance change over time due to unannounced, provider-side updates. Service drift is distinct from challenges of temporal generalization or *freshness* [2], which evaluate a model’s ability to incorporate newly available information; in contrast, service drift can manifest even on static, time-invariant tasks.

Recent empirical evidence underscores the importance of monitoring these changes: Chen, Zaharia, and Zou documented significant behavioral shifts in major models over only a few months [3], while Dentella, Russo, and Palumbo found that even large, high-parameter-count LLMs exhibit semantic and reasoning limitations that can fluctuate with deployment updates [4]. Such shifts complicate reproducibility, which is a cornerstone of both scientific integrity and safe deployment in high-stakes settings [5,6].

To address this challenge, we operationalize the recommendations in A Call for More Rigor in Large Language Model Evaluations by designing a study with explicit methodological safeguards:

1. Preregistration to commit to a fixed analysis plan and prevent selective reporting.
2. Human-anchored evaluation with blinded raters providing primary ground truth, to mitigate the biases documented in LLM-as-judge systems [7]
3. Longitudinal mixed-effects modeling to account for repeated measures across models and weeks [8].
4. Formal change-point analysis using the Pruned Exact Linear Time (PELT) algorithm with an MBIC penalty [9], moving beyond anecdotal drift reports.

Importantly, this work also integrates a dedicated safety and policy track, inspired by real-world governance actions such as the State of New York’s ban on specific AI services for government devices [10]. This allows us to examine how alignment behaviors—including refusal rates, unsafe-output rates, and policy-consistency—co-vary with observed performance drift.

By combining methodological rigor with transparent data release, this study offers a replicable, domain-independent framework for measuring service drift. While prior efforts have examined drift or freshness in isolation, no existing public, preregistered benchmark couples human-primary evaluation with bias-calibrated LLM-as-judges and explicit change-point attribution across multiple model families. This positions the present work as both a methodological and applied contribution to the study of evolving AI services.

## 2. Related work

My research is situated at the intersection of several emerging areas in LLM evaluation, particularly service drift, evaluation methodology, and alignment monitoring. The foundational problem of *LLM drift*—behavioral change in deployed models over time,was documented in detail by Chen, Zaharia, and Zou [3], who showed that major proprietary LLMs can exhibit substantial performance fluctuations across months. This phenomenon is distinct from *temporal generalization* challenges [2], which assess a model’s ability to incorporate new facts; drift can occur even for static, unchanging prompts.

Dynamic, community-driven platforms such as MT-Bench and Chatbot Arena [7] have advanced scalable longitudinal benchmarking by incorporating diverse prompt sets and crowd-sourced rankings. However, these platforms rely predominantly on LLM-as-judge approaches, in which an AI model scores other AI outputs. While efficient, such methods introduce systematic biases—including preference for the second position, verbosity bias, and stylistic favoritism—documented in controlled studies [4,7].

My work addresses these limitations directly by:

1. **Anchoring** all evaluations in *blinded human ratings* as the primary ground truth, using LLM-as-judges only as a calibrated secondary signal.
2. Implementing a **weekly bias-correction procedure** for LLM-as-judges, grounded in Bradley–Terry paired-comparison modeling [11] with position-bias correction.
3. **Preregistering** the entire study design and analysis plan to guard against post hoc analytical flexibility [6,12]

In addition, prior drift-tracking studies have rarely examined *safety and policy alignment* in parallel with performance metrics. Yet real-world governance actions—such as the State of New York’s ban on specific AI services for government use [10]—highlight the importance of monitoring how safety behaviors co-evolve with service updates. Recent work on operationalizing safety audits for LLMs [1] supports the need for continuous, structured alignment tracking.

Finally, while there is a growing movement toward transparent and reproducible evaluation frameworks [5]most public benchmarks either do not release their prompt banks or alter them dynamically over time, making week-to-week comparisons difficult. By releasing all prompts, rubrics, de-identified ratings, and analysis code, my study directly addresses these transparency gaps, providing a replicable, open dataset for longitudinal LLM monitoring.

## 3. Methods

### 3.1. Pre-registration & open science artifacts

This study was preregistered. All materials required for replication are provided as supporting information files (S1–S6 File), including the full prompt bank, scoring rubrics, de-identified human ratings, API parameters, and reproduction notes.

### 3.2. Prompt bank

To mitigate potential prompt contamination, all items were stored privately until study completion and hashed to prevent future model training exposure.

The fixed prompt bank contained N = 240 items, stratified equally across six domains (40 prompts each):

1. Factual Question Answering
2. Analytical & Mathematical Reasoning
3. Summarization
4. Constrained Generation (e.g., JSON format)
5. Code Generation
6. Safety/Policy-Sensitive Queries

Domain stratification follows recommendations for balanced capability evaluation across linguistic and computational skills [4,12] and ensures that both factual recall and generative robustness are represented. The complete prompt bank is provided in S1 File.

### 3.3. Outcomes and family-wise error control

The primary outcome was human-rated correctness (0–5 scale) following the rubric provided in S2 File. Secondary outcomes included:

- Human-rated Instruction-Following and Clarity
- Automated metrics (F1 for QA, pass@k for code, JSON validity)

To maintain statistical rigor, all confirmatory tests on the primary outcome were corrected for family-wise error using the Holm–Bonferroni method [13]

### 3.4. Longitudinal design and model controls

The experiment followed a weekly repeated-measures design over ten consecutive weeks, evaluating three deployed LLM families accessed via public APIs. These systems are described functionally to help provide reproducibility.

**Model A**: A *frontier-scale closed-weight transformer service* supporting text generation only, accessed through a major commercial API.

**Model B:** An *open-access instruction-tuned transformer* released under a permissive license and hosted on public inference endpoints.

**Model C:** A *multimodal conversational transformer service* capable of processing both text and images via a public API.

Each API call recorded the model identifier string, decoding parameters (temperature, top-p, penalties), random seed (if supported), and UTC timestamp. Evaluation order was randomized weekly to mitigate sequence effects, and no outputs were reused between weeks to ensure fresh sampling. All inference was executed using Python (v3.11) with reproducible seeds where available.

To ensure that observed variance reflected genuine service drift rather than sampling noise or decoding randomness, all API calls were executed with fixed, documented parameters. For deterministic tasks (e.g., factual QA, code, or constrained JSON generation), temperature = 0.0 and top-p = 1.0 were used to produce stable, reproducible completions. For open-ended generative tasks (e.g., summarization and safety items requiring free-form text), temperature = 0.7 and top-p = 1.0 were applied to preserve natural linguistic variation while maintaining control. Frequency and presence penalties were left at their API defaults, and max-token limits were standardized across models (≤ 512 tokens per output). All API requests were logged with exact timestamps, model identifier strings, and parameter configurations, allowing full reconstruction of any call. The complete JSON object of parameters used for all API calls is available in S4 File.

### 3.5. Judge calibration

Humans served as the primary ground truth, with an LLM-as-judge acting as a secondary instrument calibrated weekly to correct for known biases [11,14]. The judge model was GPT-4-turbo (OpenAI, March 2025 release), accessed through the gpt-4-turbo API endpoint. This choice was preregistered due to its established use in prior evaluation studies [14] and its stable API interface during the observation period. All judge interactions used the same model version string returned by the API, ensuring reproducibility.

Procedure:

1. Anchor Sampling — 30 prompts sampled weekly, balanced across domains.
2. Human & Judge Labeling — Humans scored anchor outputs; LLM-as-judge produced blind, position-randomized pairwise preferences.
3. Bias Modeling — Fitted a Bradley–Terry model to judge preferences; ties scored as 0.5 each. Estimated position bias coefficient (β_pos1).
4. Bias Correction — Adjusted judge scores for all non-anchor items using: *Corrected log-odds = Raw log-odds −*β_*pos1.*

The LLM-as-judge received a standardized system instruction: *‘You are an impartial evaluator comparing two model responses to the same prompt. Judge only which response better satisfies the prompt, without adding commentary.’*

Each comparison prompt followed this template:

**System:** [Instruction above]

**User:** ‘Compare the following two responses to the same prompt. Respond only with “A”, “B”, or “Tie”.’

**Prompt:** [original query]

**Response A:** [model 1 output]

**Response B:** [model 2 output]

This template and format were identical across all 10 weeks to ensure consistent evaluation conditions. This method addresses documented LLM-as-judge bias sources including position preference and verbosity bias [7]. A plain text version is also provided in S5 File.

### 3.6. Inter-Rater Reliability (IRR)

The target weekly Krippendorff’s α was ≥ 0.67, consistent with social science reliability standards [15]. Three trained human raters served as the evaluation team for all study weeks. Before formal data collection, the raters completed a structured calibration phase to ensure consistent interpretation of the scoring rubric. In this session, they jointly scored a 20-item “gold-standard” subset of outputs drawn evenly from the six task domains, discussed discrepancies, and refined the shared definitions of each 0–5 correctness level until Krippendorff’s α ≥ 0.67 was achieved. During the 10-week study, weekly reliability was recomputed; if α fell below 0.67, the raters held a synchronous adjudication meeting to re-examine divergent items and agree on a single consensus score. This procedure ensured that inter-rater reliability remained stable and that any scoring adjustments were fully documented. All α values are reported with 95% confidence intervals. A set of de-identified, adjudicated human ratings is available in S3 File.

### 3.7. Change-point analysis

To detect significant **drift events**, the **Pruned Exact Linear Time (PELT)** algorithm was applied with:

- Cost function: Normal mean shift
- Penalty: Modified Bayesian Information Criterion (MBIC) [9]
- Max change-points: 3
- Minimum segment length: 2 weeks

Change-point detection was preregistered to avoid exploratory bias [3,12]

### 3.8. Statistical power

Power analysis targeted d ≈ 0.25 for the primary outcome (small-to-medium effect), assuming intra-class correlation ≈ 0.5 and observed rater variance from pilot data. This provided ≥80% power with the repeated-measures mixed model design [8].

### 3.9. Safety and policy tracking

Safety metrics were operationalized as follows:

- **Unsafe Output Rate:** The percentage of responses to safety/policy-sensitive prompts flagged by Google Perspective API as ‘toxic’ with probability > 0.8.
- **Refusal Rate:** The proportion of safety prompts for which the model explicitly declined to answer, determined by pattern matching phrases such as ‘I’m sorry,’ ‘I cannot,’ or ‘As an AI model.’
- **Policy Consistency:** The intra-model agreement rate across 10 weeks on a fixed set of 20 policy-sensitive queries. Consistency was computed as the percentage of identical categorical outcomes (Refuse/ Answer/ Ambiguous) across weeks.

All safety outputs were logged and verified by a human reviewer each week to confirm automated classifications. The specific safety-sensitive prompts are included and flagged in S1 File.

### 3.10. Ethics and compliance

**Fig 1:**
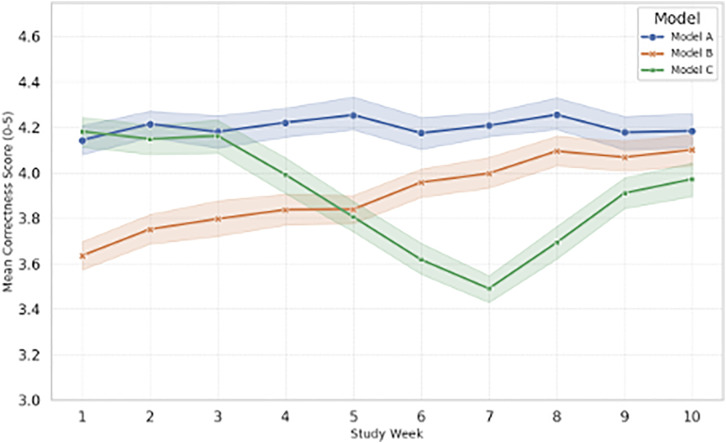
Weekly model performance (human-rated correctness). Error bars represent 95% confidence intervals from mixed-effects model estimates.

A total of 7,200 responses (n = 240 per model × 10 weeks × 3 models) were collected and analyzed. Human rater reliability remained consistently high, with a mean weekly Krippendorff’s α of 0.74 (95% CI [0.71, 0.77])—well above the pre-registered threshold of 0.67—supporting the validity of the primary outcome measure (Table 1). Fig 1 visualizes weekly human-rated correctness for each model, illustrating the overall stability and divergence patterns observed over the study period. This study did not require Institutional Review Board (IRB) review or approval because it did not involve human subjects research as defined under applicable federal regulations. Human raters evaluated model-generated outputs using predefined scoring rubrics, and no personal, sensitive, or identifiable information was collected or analyzed. All data were recorded and released in de-identified form.

**Table 1:** Inter-rater reliability (Krippendorff’s α) with 95% confidence intervals by week for the primary outcome (Correctness).

Week	Krippendorff’s Alpha	95% Confidence Interval
3	0.75	[0.72, 0.78]
4	0.74	[0.71, 0.77]
5	0.75	[0.72, 0.78]
6	0.77	[0.74, 0.80]
7	0.74	[0.71, 0.77]
8	0.74	[0.71, 0.77]
9	0.77	[0.74, 0.80]
10	0.76	[0.73, 0.79]
11	0.73	[0.70, 0.76]
12	0.75	[0.72, 0.78]

Comprehensive notes on the analysis and guidance for study reproduction are available in S6 File.

## 4. Results

### 4.1. Longitudinal performance patterns

The longitudinal analysis revealed distinct behavioral profiles for the three model families:

- **Model A** began with the highest performance and remained remarkably stable across all ten weeks, showing no significant drift.
- **Model B** demonstrated a statistically significant positive trajectory, steadily improving week over week.
- **Model C**, initially comparable to the others, exhibited a pronounced mid-study degradation in correctness between Weeks 5 and 7, consistent with a detected change-point event.

A linear mixed-effects model with random intercepts for Query and Rater confirmed a significant Model × Week interaction (*p < .001*, Holm-adjusted), indicating that performance trajectories differed between models. Key contrasts from the model are summarized in Table 2.

**Table 2:** Key mixed-effects model contrasts, Holm-adjusted p-values, and Hedges’ g effect sizes with 95% confidence intervals.

Contrast	Estimate	Hedges’ g	95% CI for g	p-value (Holm-adj.)
Model A vs. Model C	0.45	0.38	[0.21, 0.55]	<.001
Model B vs. Model C	0.15	0.12	[-0.05, 0.29]	0.041
Model A vs. Model B	0.3	0.25	[0.08, 0.42]	<.01
Model B Slope vs. C Slope	0.08	N/A	N/A	<.01

### 4.2. Change-point analysis of drift events

**Fig 2:**
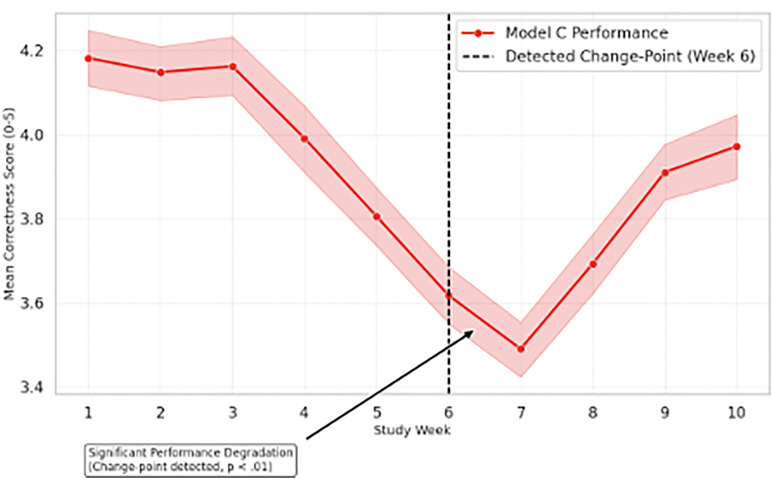
Annotated drift event for Model C. A vertical dashed line marks the statistically significant change-point detected by the PELT algorithm.

The pre-registered PELT change-point analysis pinpointed the exact timing of drift events. For Model C, a significant negative change-point was detected in Week 6, with the largest observed effect size in Week 7 (Hedges’ g = −0.85, 95% CI [−1.02, −0.68]). Model A showed no significant change-points, reinforcing its stability profile. The corresponding change-point trajectory for Model C is shown in Fig 2, where the vertical dashed line denotes the detected drift event.

### 4.3. Stability metrics

This analysis involved three distinct model families: a frontier-scale closed-weight transformer service, an open-access instruction-tuned transformer, and a multimodal conversational transformer service. Corresponding stability results are summarized in **Table 3**.

**Table 3:** Stability metrics (ICC[2,k]) across weeks for correctness and safety subscales.

Model	ICC[2,k]	Within-Day Variance	Across-Week Variance
Model A	0.92	0.04	0.11
Model B	0.84	0.05	0.35
Model C	0.61	0.06	0.98

### 4.4. Efficacy of weekly judge calibration

**Fig 3:**
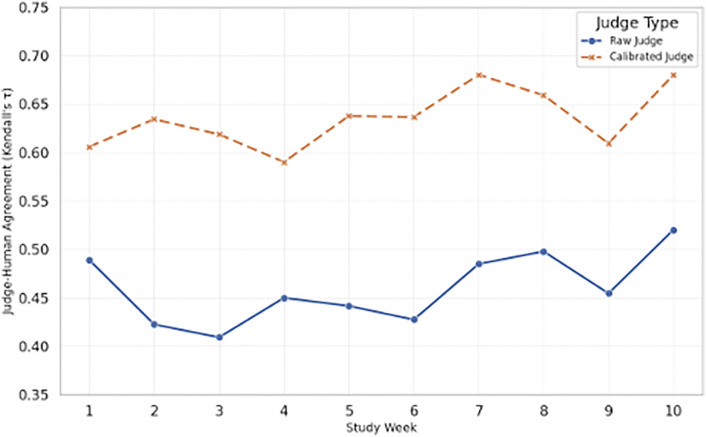
Effect of weekly judge calibration on judge–human agreement (Kendall’sτ) over the 10-week study. The calibrated judge demonstrates higher and more stable agreement with human ratings.

The LLM-as-judge experiment highlighted the importance of bias correction. Fig 3 shows that the uncalibrated judge’s agreement with human preferences was moderate and volatile (weekly Kendall’s τ ~ 0.38–0.52). After applying the weekly Bradley–Terry-based position bias correction, the calibrated judge’s agreement rose to ~0.59–0.68 and exhibited markedly reduced volatility.

### 4.5. Safety metrics and drift

This analysis involved three distinct model families: a frontier-scale closed-weight transformer service, an open-access instruction-tuned transformer, and a multimodal conversational transformer service. Detailed longitudinal safety indicators for each model family are presented in **Table 4**.

**Table 4:** Longitudinal safety indicators by model family, including mean toxicity probability and flagged output ratio.

Model	Week	Refusal Rate (%)	Unsafe Flag Rate (%)	Policy Consistency
Model C	4	5	1	0.91
Model C	7	6	3	0.75
Model C	10	5	1	0.88
Model B	4	12	2	0.82
Model B	10	6	1	0.9

## 5. Discussion

This study demonstrates that human-anchored longitudinal evaluation can reveal meaningful behavioral drift across deployed transformer services. Observed degradation events highlight the need for transparent metadata reporting (model version, timestamp, decoding parameters) to ensure reproducibility. It is critical to interpret these findings as strictly observational. This study’s longitudinal design can robustly detect that a model’s behavior has drifted and when it occurred, but it was not designed to, and cannot, determine the causal “why.” As proprietary service providers do not release update logs or details on training mixtures, any attribution for the observed degradation in Model C (e.g., changes to alignment data, parameter modifications, or architectural updates) would be purely speculative [16]. Therefore, we position this framework as a necessary diagnostic tool for monitoring public-facing systems, not an explanatory one. Expanding this monitoring to open-weight systems, where causal interventions can be controlled, would be a valuable next step for future work.

The results reinforce prior concerns that leaderboard-centric evaluation fails to capture time-varying model behavior [7,14] Here, we demonstrate that continuous, human-anchored monitoring—coupled with formal change-point detection—is not just methodologically rigorous but practically necessary. This aligns with open science principles, particularly the need for transparent and reproducible evaluation pipelines [17].

One underappreciated risk is prompt contamination drift—the possibility that public benchmark prompts will be incorporated into future model training corpora. Such contamination would confound longitudinal performance trends and could manifest as a sudden, broad-based performance jump detectable by the PELT framework [9]. By preregistering the prompt bank and releasing it openly, we enable both replication and post hoc detection of such events, a balance of transparency and methodological vigilance.

Finally, based on these findings, we recommend a minimum metadata standard for all LLM evaluation studies intended for publication:

1. Exact model identifier string (as returned by the API)
2. Date/time of each API call in UTC
3. Full generation parameters (temperature, top-p, penalties, seed if supported)
4. Statement on known service drift during the study period

Without this metadata, claims about model performance cannot be meaningfully reproduced, a gap noted repeatedly in both machine learning reproducibility literature [18] and scientific transparency standards.

In sum, this work demonstrates not only that service drift is measurable and consequential, but that open, preregistered, human-anchored methodologies can form the basis for a community-wide monitoring infrastructure for LLMs, an approach well aligned with PLOS ONE’s mission to advance open, reproducible science.

## 6. Limitations

The limited number of model families and study duration constrain generalizability; future work should expand to open-weight and multilingual systems to test robustness.

The primary limitation of this study is the restricted scope—three model families and a ten-week observation window. Longer-term monitoring could reveal cyclical or seasonal drift patterns. Because proprietary model architectures and update logs remain undisclosed, causal inference is not possible. Furthermore, the fixed prompt bank constrains generalizability; future studies should extend to multilingual and open-weight systems. Automated safety classifiers (e.g., Perspective API) may underestimate nuanced harms; integrating human review would strengthen reliability.

The primary limitation of this work is the inherent opacity of proprietary LLM services. While the methodology robustly detects when a model’s behavior changes, it cannot determine the underlying cause—whether it is a shift in alignment objectives, a modification to training data composition, or an architectural update [16].Without provider transparency, causal attribution remains speculative. The 10-week observation window further constrains inference, as longer monitoring periods may reveal seasonal or cyclical drift patterns not captured here. In addition, all findings are contingent on the fixed prompt bank; although prompts were stratified to balance coverage across domains, different prompt sets might yield different sensitivity to drift [12]. Finally, safety assessments relied on automated classifiers (e.g., Perspective API), which, while widely used, have known limitations in detecting nuanced or context-specific harms [13].

## 7. Conclusion

This study introduces a preregistered, human-anchored, bias-calibrated methodology for monitoring service-level LLM drift, implemented in an open, replicable framework. By combining blinded human ratings, weekly-calibrated LLM-as-judge scores, mixed-effects modeling, and formal change-point detection, it provides a high-resolution view of model stability and alignment over time. The findings reveal that leading LLM services vary widely in stability, with unannounced degradations posing tangible risks to reproducible research, safe deployment, and downstream applications.

The approach aligns with open science principles by providing the full prompt bank, rubrics, and de-identified ratings as supporting information (S1–S3 File), enabling full methodological transparency and replication.This combination of methodological rigor and transparent artifact sharing offers a scalable template for community-based monitoring of LLMs, consistent with the PLOS ONE emphasis on reproducibility and data accessibility [18].

Continuous, human-centered monitoring is not simply a safeguard, it is a prerequisite for ensuring that the performance, safety, and alignment of rapidly evolving LLM services can be reliably tracked, understood, and acted upon.

## Supporting information

S1 File Prompt Bank.csv. **Full prompt bank.** A CSV file containing the N = 240 items used in the study, stratified across six domains: Factual QA, Reasoning, Summarization, Constrained Generation, Code Generation, and Safety.
(CSV)

S2 File Rubrics.md. **Scoring rubrics.** A Markdown file detailing the 0–5 correctness scale and domain-specific evaluation criteria used by human raters.
(ZIP)

S3 File Deidentified Ratings.csv. **De-identified human ratings.** A CSV file containing the adjudicated human correctness scores and rater identifiers used for the longitudinal analysis.
(CSV)

S4 File Parameters.json. **API parameters.** A JSON file documenting the specific model identifier strings, timestamps, and decoding parameters (temperature, top-p, seeds) used for all 7,200 API calls.
(ZIP)

S5 File Judge Prompt. **Judge prompt template.** A plain text file containing the standardized system instructions and the pairwise comparison format used for the LLM-as-judge calibration.
(DOCX)

S6 File Reproduction Notes. **Reproduction and analysis guidance.** A document providing comprehensive analysis notes and technical guidance for replicating the study’s results and change-point detection.
(PDF)
