# Supplementary material for: Human-anchored longitudinal comparison of generative AI with a bias-calibrated LLM-as-judge
Source: PLoS One. 2026 Feb 2;21(2):e0339920. doi: 10.1371/journal.pone.0339920 (PMC12863567; doi:10.1371/journal.pone.0339920)
Supplement: S2 File — Scoring rubrics. A Markdown file detailing the 0–5 correctness scale and domain-specific evaluation criteria used by human raters. (ZIP) [file pone.0339920.s002.zip › S2/S2_Rubrics.pdf]

Official Scoring Rubrics for Longitudinal LLM Study

Correctness

Score	Description
5	Completely accurate and logically sound.
4	Mostly correct; minor inaccuracies.
3	Main idea correct; noticeable errors.
2	Mostly incorrect; fundamentally flawed.
1	Entirely incorrect or nonsensical.
0	No response or unrelated.

Instruction-Following

Score	Description
5	Perfect adherence to instructions and format.
4	Minor deviation on trivial constraint.
3	Follows main instruction but misses key constraint.
2	Attempts answer but ignores most constraints.
1	Ignores instructions entirely.
0	No response.

Clarity

Score	Description
5	Exceptionally clear and well-written.
4	Clear with minor issues.
3	Understandable but awkward or error-prone.
2	Difficult to follow or disorganized.
1	Incoherent.
0	No response.
