# Supplementary material for: Human-anchored longitudinal comparison of generative AI with a bias-calibrated LLM-as-judge
Source: PLoS One. 2026 Feb 2;21(2):e0339920. doi: 10.1371/journal.pone.0339920 (PMC12863567; doi:10.1371/journal.pone.0339920)
Supplement: S6 File — Reproduction and analysis guidance. A document providing comprehensive analysis notes and technical guidance for replicating the study’s results and change-point detection. (PDF) [file pone.0339920.s006.pdf]

Supporting Information S6 – Reproduction Notes

Supporting Information S6 – Reproduction Notes

Environment Setup
- Python version: 3.11
- Dependencies: pandas, numpy, statsmodels, ruptures, scikit-learn
- Operating system: macOS 14 / Ubuntu 22.04
- All analyses were run on a local environment with CPU execution only.

Data Structure and Files
- S1_Prompt_Bank.csv – Fixed prompt bank of 240 items.
- S2_Rubrics.md – Human scoring rubric definitions.
- S3_Deidentified_Ratings.csv – Human ratings (de-identified).
- S4_Parameters.json – Inference parameters and model metadata.
- S5_Judge_Prompt.txt – Evaluation prompt for LLM-as-judge calibration.

Analysis Pipeline
1. Human ratings were aggregated weekly using mixed-effects models (random intercepts for Query and Rater).
2. Change-point analysis used the Pruned Exact Linear Time (PELT) algorithm with MBIC penalty.
3. Inter-rater reliability computed using Krippendorff's α (bootstrap 95% CIs).
4. Safety metrics (Refusal Rate, Unsafe Output Rate, Policy Consistency) were computed from flagged responses.

Verification and Reproducibility
- All datasets and scripts are reproducible with public artifacts (S1–S5).
- Proprietary model outputs are not redistributed, but all hashes and calibration coefficients are included.
- The full analysis can be replicated using the same prompt bank and scoring rubrics with any compliant LLM API.

Final Note
All analyses can be reproduced using the data and rubrics provided (S1–S5).
Proprietary model outputs are not redistributed; however, all hash signatures and calibration coefficients
are included for verification.
